# A Case of Toxoplasmic Encephalitis in an HIV-Negative Patient With Multiple Cerebral Infarction-Like Findings in the Early Stages

**DOI:** 10.7759/cureus.83603

**Published:** 2025-05-06

**Authors:** Ken Sakamoto, Yoshiaki Nakayama, Shuhei Ogami, Yusuke Koizumi, Katsuichi Miyamoto

**Affiliations:** 1 Department of Neurology, Wakayama Medical University, Wakayama, JPN; 2 Department of Infection Control and Prevention, Wakayama Medical University, Wakayama, JPN

**Keywords:** atypical imaging findings, atypical mri findings, immunocompromised host, non-hiv-infected patients, toxoplasmic encephalitis

## Abstract

Toxoplasmic encephalitis (TE) is an opportunistic infection that typically manifests in immunocompromised individuals, such as those with HIV infection or AIDS. The characteristic imaging finding is a mass-like lesion with ring enhancement. We report a rare case of TE in a patient who was not infected with HIV with atypical imaging features. An 81-year-old woman developed progressive impaired consciousness over several weeks. Initially, the brain MRI revealed multiple lesions resembling cerebral infarctions, which gradually enlarged and increased in number. Subsequently, gadolinium-enhanced MRI showed ring-enhancing mass-like lesions in the pons and left parietal lobe. Blood tests indicated elevated inflammatory markers and a mildly increased *Toxoplasma *IgG level. Given the progressive course, empiric therapy with trimethoprim-sulfamethoxazole and clindamycin was initiated, leading to marked improvement in consciousness by the fourth day. *Toxoplasma *DNA was later detected in the blood, confirming the diagnosis of TE.

## Introduction

Toxoplasmic encephalitis (TE) is an opportunistic infection most commonly seen in immunocompromised individuals, particularly those with HIV/AIDS, where reactivation of latent *Toxoplasma gondii *occurs due to impaired immunity [1, 2]. The typical radiological hallmark is a mass-like lesion with ring enhancement on CT or MRI, which is critical for diagnosis [3, 4]. In contrast, TE in patients not infected with HIV is rare, and cases presenting with infarction-like lesions on imaging are even more uncommon. We describe a case of TE in an 81-year-old woman with a history of immunosuppressive therapy who presented with multiple cerebral infarction-like findings during the early disease phase. This case contributes valuable insight into the variable imaging presentations of TE in non-HIV immunocompromised individuals.

## Case presentation

**Figure 1:**
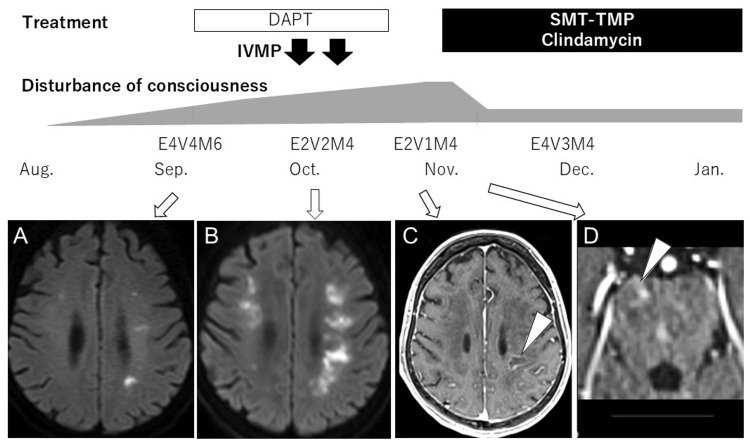
The patient's clinical course and MRI findings In August, the patient developed fatigue and loss of appetite. In September, she presented with a fever, and her level of consciousness declined to a Glasgow Coma Scale (GCS) score of E4V4M6. Brain MRI revealed multiple lesions in the deep white matter of the middle cerebral artery territory (A). The lesions were suspected to be multiple cerebral infarcts, and dual antiplatelet therapy (DAPT) was initiated; however, her level of consciousness continued to deteriorate. Subsequently, mass-like lesions with ring-shaped gadolinium enhancement appeared (arrowheads) in the left parietal lobe (C) and pons (D), raising suspicion for toxoplasmic encephalitis. Treatment with a combination of sulfamethoxazole (SMX)-trimethoprim (TMP) and clindamycin was initiated, leading to an improvement in her consciousness level to a GCS score of E4V3M4. As her condition remained stable, she was transferred to a rehabilitation hospital in January of the following year. (A) and (B): Diffusion-weighted images; (C) and (D): Gadolinium-enhanced T1-weighted images with fat suppression. Aug: August 2024; IVMP: intravenous pulse methylprednisolone

An 81-year-old woman, previously independent in activities of daily living, presented with progressive fatigue and reduced oral intake from mid-August 2024. Her medical history included anti-aminoacyl-tRNA synthetase (ARS) antibody syndrome and aortic stenosis. Medications included prednisolone (5 mg/day), azathioprine (50 mg/day), and clopidogrel (75 mg/day). She had no history of allergies, alcohol consumption, or smoking and did not own pets or travel overseas. In early September, she developed a fever and altered consciousness, prompting admission to a previous hospital. Brain MRI revealed multiple infarction-like lesions in the middle cerebral artery territory (Figure 1A), and dual antiplatelet therapy was initiated; however, her neurological status deteriorated. Cerebrospinal fluid (CSF) analysis revealed elevated protein (160 mg/dL) and increased mononuclear cells (14/μL), raising suspicion for encephalitis. Two courses of steroid pulse therapy (1 g/day for 3 days) were administered without improvement, and follow-up MRI showed worsening lesions (Figure 1B). She was subsequently transferred to our hospital in October.

On admission, her Glasgow Coma Scale (GCS) score was E2V2M4. Vital signs were stable, and no signs of meningeal irritation were observed. Neurological examination showed no cranial nerve deficits. Motor assessment was limited due to impaired consciousness, though muscle strength appeared preserved. Tendon reflexes were normal; however, the left Chaddock reflex was positive. No significant sensory deficits were noted. The initial MRI showed multiple infarction-like lesions, which progressively enlarged and increased in number. Gadolinium-enhanced MRI later revealed ring-enhancing mass-like lesions in the pons and left parietal lobe (Figure 1C, 1D). Blood test findings showed a slight decrease in hemoglobin (Hb) and platelet count. In the general biochemical tests, albumin was decreased, gamma-glutamyl transpeptidase (γ-GTP) was slightly elevated, and sodium (Na) and potassium (K) levels were decreased. The ammonia level was normal, but thyroid hormone (free T4) was slightly decreased. Serum angiotensin-converting enzyme (ACE) was normal. Anti-aquaporin-4 antibody was negative. Tests for (1→3)-β-D-glucan, *Cryptococcus neoformans* antigen, Aspergillus antigen, CMV antigenemia (C7-horseradish peroxidase: C7-HRP), and T-cell assay for tuberculosis were all negative. For toxoplasmosis, serum IgM was negative, and IgG was mildly elevated at 2.1 IU/mL (reference range: <1.6) (Table 1). CSF analysis revealed elevated protein (187 mg/dL; reference: 15-45), mildly increased mononuclear cells (4/μL; reference: ≤2), and decreased glucose (59 mg/dL; with simultaneous blood glucose of 178 mg/dL). CSF ACE level was normal. All additional tests, including bacterial and mycobacterial cultures, India ink staining, John Cunningham virus (JCV)-DNA, Epstein-Barr virus nucleic acid quantification, *Candida mannan* antigen, and *Aspergillus galactomannan* antigen, were negative (Table 2). Due to the progressive clinical course and supporting lab findings, TE was suspected. Empiric treatment with sulfamethoxazole-trimethoprim and clindamycin was initiated [5]. By the fourth day, her GCS score improved to E4V3M4. *Toxoplasma *DNA was subsequently detected in the blood sample, confirming the diagnosis. Her condition continued to improve with treatment, and she was transferred to a rehabilitation facility in the second month of hospitalization.

**Table 1:** The patient's blood test reports

Parameters	Patient values	Reference range
White blood cell (WBC)	5180 /μL	3300–8600 /μL
Red blood cel (RBCl)	285 ×10⁴ /μL	427–570 ×10⁴ /μL
Hemoglobin (Hb)	9.4 g/dL	11.6-14.8 g/dL
Platelets	12.8 ×10⁴ /μL	15.8–34.8 ×10⁴ /μL
Albumin	2.8 g/dL	4.1–5.1 g/dL
Aspartate transaminase (serum glutamic-oxaloacetic transaminase (SGOT))	27 U/L	13–30 U/L
Alanine aminotransferase (serum glutamic-pyruvic transaminase (SGPT))	16 U/L	7–24 U/L
Gamma-glutamyl transpeptidase, (γ-GTP)	44 U/L	9–32 U/L
Blood urea nitrogen (BUN)	18.8 mg/dL	8–20 mg/dL
Creatinine	0.50 mg/dL	0.46–0.79 mg/dL
Glucose	79 mg/dL	73-109 mg/dL
Sodium (Na)	131 mEq/L	138–145 mEq/L
Potassium (K)	2.8 mEq/L	3.6–4.8 mEq/L
Calcium	8.8 mg/dL	8.8–10.1 mEq/L
Ammonia (NH_3_)	29 μg/dL	30-80
Free thyroxine (T4)	0.81 ng/dL	0.90-1.70
Angiotensin-converting enzyme (ACE)	8.7 U/L	7-25 U/L
Anti-aquaporin-4 antibody	<1.5 U/mL	<3.0 U/mL
(1→3)-β-D-glucan	<6 pg/mL	0-11 pg/mL
*Cryptococcus neoformans* antigen	negative	negative
*Aspergillus *antigen	Index <0.2	Index <0.5
C7–horseradish peroxidase	negative	negative
T-cell assay for tuberculosis	negative	negative
Toxoplasmosis, IgM	＜0.8 IU/mL	＜0.8 IU/mL
Toxoplasmosis, IgG	2.1 IU/mL	<1.6

**Table 2:** The patient's cerebrospinal fluid (CSF) test reports

Parameters	Patient values	Reference range
Opening pressure	10 mmH₂O	7–18 mmH₂O
Cell count	4 /μL	0–2 /μL
Protein	187 mg/dL	15–45 mg/dL
Glucose	59 mg/dL	60–70% of blood glucose
Angiotensin-converting enzyme (ACE)	0.3 U/L	<1.0 U/L
Bacterial and mycobacterial cultures	Negative	Negative
India ink staining	Negative	Negative
John Cunningham virus (JCV)-DNA	Negative	Negative
Epstein–Barr virus nucleic acid quantification	Negative	Negative
*Aspergillus galactomannan* antigen	Negative	Negative

## Discussion

TE usually presents as mass-like brain lesions with ring enhancement on MRI or CT and may also involve ventriculitis [6, 7]. In non-HIV-infected patients, TE often presents as single or multiple nodular or ring-enhancing lesions, commonly at the gray-white matter junction with surrounding subcortical edema [8]. In this case, however, the initial findings were atypical, resembling multiple cerebral infarctions. The patient's advanced age and vascular risk factors initially made infarction a more plausible diagnosis. However, the emergence of ring-enhancing lesions with gadolinium enhancement led to consideration of TE.

Pathologically, TE is characterized by vascular occlusion and necrosis of vessel walls and surrounding tissues [9]. The infarct-like MRI findings in this case may reflect these underlying vascular changes. Additionally, CSF and blood tests were crucial in guiding the diagnosis. Notably, the detection of *Toxoplasma* DNA in the blood was decisive in confirming TE.

TE in patients who are not infected with HIV is rare and often difficult to diagnose. The use of immunosuppressive drugs is a risk factor for the development of TE. There have been reports of rheumatoid patients undergoing treatment with methotrexate and infliximab who developed cranial nerve palsy and gait disturbance and were diagnosed with TE by brain biopsy [10], and a report of a patient with myasthenia gravis who was undergoing long-term mycophenolate mofetil treatment developing TE [11]. These reports suggest the importance of considering TE in the differential diagnosis when neurological symptoms appear in patients receiving immunosuppressive therapy. Therefore, as in our case, this highlights the importance of including TE in the differential diagnosis of immunocompromised patients, even when neurological symptoms and imaging findings are atypical for TE [12]. Previous reports suggest that treatment response in non-HIV TE is generally favorable [8], consistent with the marked improvement observed in this case after initiating appropriate therapy.

## Conclusions

We report a rare case of TE presenting with multiple infarct-like lesions in the early phase. In immunosuppressed patients with neurological symptoms and atypical imaging findings, infectious etiologies such as TE should be considered. Prompt initiation of anti-*Toxoplasma *therapy can significantly improve clinical outcomes, even in the absence of classic imaging features.
